# Singultus as an Unusual Debut of Plasmodium vivax Malaria

**DOI:** 10.7759/cureus.5548

**Published:** 2019-09-01

**Authors:** Francisco Guadarrama-Conzuelo, Assad D Saad Manzanera

**Affiliations:** 1 Escuela De Medicina, Instituto Tecnológico Y De Estudios Superiores De Monterrey, Monterrey, MEX; 2 Psychiatry, National Institute of Psychiatry Ramón De La Fuente Muñiz, Mexico City, MEX

**Keywords:** singultus, plasmodium vivax, malaria, severe malaria, plasmodium, hiccup, undiagnosed fever

## Abstract

Singultus is a common symptom with usually benign consequences. Although some rare associations with severe diseases have been described, no previous relationship between singultus and *Plasmodium vivax* malaria has been reported. The aim of this report is to detail the case of a 25-year-old male who had an unusual presentation for both singultus and laboratory-confirmed *vivax* malaria. We describe this previously unreported association, hypothesize on the potential mechanism and review the literature on singultus and unusual presentations of *Plasmodium* infections.

## Introduction

Hiccups (or singultus, as they are also known) are spontaneous contractions of the diaphragm and intercostal muscles often considered to be a bothersome, common, trifling symptom that usually resolves without medical interventions [1]. With a yet unclear physiological function in adults [2], they are thought to be the consequence of a reflex arc that is the result of over-distension of the gastric chamber or the exposure to irritants of either the respiratory or the gastrointestinal tracts [3]. While its most frequent presentation is that of a self-limiting, short-lived discomfort of little importance, in some rare instances singultus has been associated with more ominous diseases with nefarious outcomes [4].

Although previously reported as a consequence of the administration of doxycycline for the treatment of *Plasmodium falciparum* [5], this is -to our knowledge- the first time that persistent singultus has been the debuting symptom of *vivax* malaria.

Malaria (a word derived from the medieval Italian *mala aria*, “bad air”) is a widespread parasitic disease of temperate climates [6]. After a few days, typical malaria symptoms develop and the patients often experience very high fever, malaise, fatigue, headache, diaphoresis, chills, diarrhoea, mild abdominal pain, and nausea [7]. Neurological symptoms may be present in a rarer, more severe form of the disease known as cerebral malaria [8].

Here, we report the case of a 25-year-old male who had an unusual presentation for both singultus and laboratory-confirmed *Plasmodium vivax* malaria and describe this previously unreported association.

## Case presentation

A 25-year-old previously healthy male presented for evaluation for singultus. He refers to have self-treated his symptoms, initially with prokinetics and antacids, and after seeing no improvement, with an oral benzodiazepine. As he found no resolution of the symptoms, he sought specialized medical care.

A comprehensive medical history was obtained. He reported having primary isolated hypertriglyceridemia and was otherwise healthy with no family history of neurodegenerative or autoimmune diseases. His father suffers from primary hypertension as did his paternal grandparents. One non-immediate-family member was diagnosed with HER2+, BCRA-negative breast cancer and another one died of type-2 diabetes complications. The patient reported no previous exposure to hazardous chemicals, drugs, ethanol, recent surgical or dental interventions. He had received all the Centers for Disease Control (CDC) recommended immunizations for healthcare personnel and the initial dose of the tetravalent recombinant Dengue vaccine (Dengvaxia®).

For the previous twelve months, the patient had been working as a primary-care physician in a marginalized mountainous region located near the Mexico-Guatemala border. He did not recall having treated anyone for neurological infections or anything he thought as being “out of the ordinary”. He had regular contact with poultry, cows, deer, cats, and dogs. Boiled water was available.

About thirty days before his symptoms began, he had traveled to the Lacandon jungle in southern Mexico, where he was in close contact with exotic birds, crocodiles, bats, turtles, freshwater fish, frogs and a plethora of varied tropical insects. At the time, he was not taking any medicines and did not experience any symptoms.

He informed the medical team that his symptoms had begun approximately 27 hours prior to his consultation, when he developed hiccups the previous morning. About 7 or 8 hours after the hiccups had begun, he noted the increased frequency of his hiccups and self-prescribed 10mg metoclopramide and 20mg omeprazole, and performed a few physical maneuvers customarily used in the treatment of hiccups (breath-holding and Valsalva maneuver). Later, he took a second dose of metoclopramide 6 hours after the first one. Growing in despair, as he had been suffering from singultus for about 18 hours, he took a 1mg lorazepam tablet in the hopes that it would end his hiccups and let him sleep. He went to bed and woke from troubled sleep about 5 hours later.

When he woke up, he refers that although the singultus frequency had slowed, the symptom not only persisted but it was now accompanied by dizziness. He attributed the lightheadedness to the lorazepam tablet but became alarmed once he discovered that he had developed binocular diplopia, nausea and gait disturbances. He sought immediate medical consultation at a teaching hospital. 

At the emergency room (ER), his vitals were within reference parameters: blood pressure (BP) was 118/76 mmHg, heart rate (HR) 88x’, respiratory rate (RR) 19x’, SatO_2_ 98% and temperature 36.6ºC (axillary). Physical examination was performed by an experienced neurologist. It showed no alterations in mental status (level of consciousness, attention, concentration, memory, language, visual and spatial perception, executive functioning, mood and thought content, praxis, and calculations). Cranial nerve (CN) examination showed no alterations in visual fields. No changes in visual acuity were detected. Funduscopic examination was unremarkable, but the patient reported to have developed photophobia. Pupillary light reflexes were normal. No eyelid ptosis was observed. Evoked jerk horizontal nystagmus was found. Facial sensation was unaltered. Corneal reflex was not tested. Right CN VI palsy was observed. CN VII examination showed slight facial asymmetry with left lower lip ptosis. His hearing was unaffected. No dysarthria or changes in tongue movement were present. Head rotation and shoulder elevation were unaltered. Strength was evaluated using the Medical Research Council Scale (MRCS): the deltoid, biceps, triceps, extensor carpi radialis, abductor pollicis brevis, interossei were found to be 5/5, bilaterally. Iliopsoas, quadriceps, hamstrings, tibialis anterior and gastrocnemius 4/5 in the left side and 5/5 in the right side. Reflexes were normal (2/4) at the biceps and triceps, bilaterally. Hyperreflexia without clonus (3/4) was found at the left knee and ankle while those on the contralateral side were normal (2/4). Plantar responses were flexor. Peripheral sensory examination was unaltered for light touch, pain, temperature, vibration, graphesthesia, stereognosis, and two-point discrimination. There was no dysmetria on finger-to-nose and heel-knee-shin. No dysdiadochokinesia was found. Rapid alternating movements and fine finger movements were intact. Romberg’s sign was present. Postural instability was found when the patient was asked to stand and close his eyes, leaning to both sides, alternatively. When asked to walk on toes and heels, gait imbalance was found. No nuchal rigidity was found. Ear examination was normal. No skin changes, abnormal heart or lung sounds were detected. The spleen was not palpable and the liver was not enlarged. The rest of the physical exam was unremarkable but singultus was observed throughout the process. The patient referred diplopia and moderate hyperacusis without tinnitus. A summary of the physical exam findings can be seen in Table 1.

**Table 1:** Summary of physical exam findings

Summary of relevant physical exam findings
Vitals within reference parameters
No alterations in mental status
No changes in visual acuity or abnormal funduscopic examination
Photophobia
Evoked jerk horizontal nystagmus
Abducens nerve palsy (limitation of abduction of the right eye)
Unaffected hearing, no dysarthria
Slight facial asymmetry, lower left lip ptosis
Reduced strength in some muscles of the left leg: (iliopsoas, quadriceps, hamstrings, tibialis anterior and gastrocnemius)
Hyperreflexia at the left knee and ankle
Romberg’s sign present
Postural instability and gait imbalance
No abnormal cutaneous, cardiac or respiratory findings
Singultus, diplopia and hyperacusis

As the physical exam showed abducens nerve palsy, photophobia, facial asymmetry, differing reflexes, postural instability and uneven strength, a contrast-enhanced brain magnetic resonance (MRI) and comprehensive blood assessments were ordered. The imaging studies did not show any abnormalities and blood tests were deemed to be unremarkable. Relevant parameters can be seen in Table 2 and Table 3. 

**Table 2:** Complete blood counts Table shows serialized assessments of haematologic parameters. A sharp decline in haemoglobin concentration can be seen throughout the course of the disease, reaching its lowest concentration by day 20. Full recovery can be seen at the 120-day follow-up.

Complete blood counts									
Parameter	Result							Units	Reference values
	Day sampled								
	1	2	8	10	13	20	120	days	-
Leukocytes	5.7	5.4	4.7	5.0	6.1	5.2	5.7	x 10^3^ /µL	4.5 - 11.0
Red-blood cells	6.5	6.3	4.9	4.48	4.17	3.8	6.15	x 10^6^ / µL	4.2 - 5.8
Haemoglobin	18	17.8	14.1	13.1	11.7	8.1	17.4	g/dL	13.2 - 18.0
Hematocrit	54	53	43	39	34.6	24.6	52	%	38.4 - 52.4
Mean corpuscular volume	83.1	83	87.8	87.2	83	88	84.6	fL	82.0 - 98.0
Mean corpuscular haemoglobin concentration	27.6	27.1	32.8	33.5	33.8	34	33.5	pg	27.0 - 31.0
Red-cell distribution width	12.7	12.6	14.5	13.6	15.6	18.4	13.7	%	11.7 - 15.5
Platelets	150	155	60	73	149	230	235	x 10^3^ /µL	150 - 420
Lymphocytes	15.2	17	23	28	35	38	42.1	%	20.0 - 40.0
Monocytes	10.8	13	2	5	1	4	7	%	3.3 - 13.3
Eosinophils	0	0	2	0	0	0	0.9	%	1.0 - 5.0
Basophils	0.5	0	0	0	0	0	0.7	%	0.0 - 1.0
Neutrophils	73.4	71.3	73	67	64	70	47.9	%	50.0 - 70.0
Absolute neutrophil count	4.2	3.8	3.4	3.5	3.9	3.64	2.75	x 10^3^ /µL	2.5 - 7.0
Absolute lymphocyte count	0.9	0.91	1.08	1.4	2.13	1.97	2.41	x 10^3^ /µL	1.0 - 4.0
Absolute monocyte count	0.6	0.7	0.94	0.25	0.06	0.2	0.40	x 10^3^ /µL	0.2 - 1.2
Absolute eosinophil count	0	0	0.94	0	0	0	0.05	x 10^3^ /µL	0.0 - 0.5
Absolute basophil count	0	0	0	0	0	0	0.04	x 10^3^ /µL	0.0 - 1.0
Method: flow cytometry with volume, conductivity and light scatter.									

**Table 3:** Complete metabolic panel assessments Summary of relevant changes in the complete metabolic panels.

Complete metabolic panel					
Parameter	Result			Units	Reference values
	Days				
	1	13	120		
Glucose	110	99	100	mg/dL	60 - 100
Creatinine	1.3	1.0	1.2	mg/dL	0.7 - 1.3
BUN (Blood urea nitrogen)	19.3	14.6	15.1	mg/dL	8.9 - 25.7
Urea	41.3	44	46	mg/dL	10.7 - 49.2
Uric acid	7.9	6.0	7.3	mg/dL	3.5 - 7.2
Cholesterol	159	67	177	mg/dL	130 - 200
Triglycerides	445	299	449	mg/dL	35 - 150
Total Calcium	9.7	8.4	9.9	mg/dL	8.4 - 10.2
Phosphorus	4	3.1	3.6	mg/dL	2.3 - 4.7
Total bilirubin	1.23	1.4	0.89	mg/dL	0.20 - 1.20
Direct bilirubin	0.4	0.83	0.21	mg/dL	0.00 - 0.50
Indirect bilirubin	0.83	0.57	0.68	mg/dL	0.10 - 0.70
Albumin	4.1	3.1	4.88	g/dL	3.5 - 5.0
Total proteins	7	5.6	7.62	g/dL	6.4 - 8.3
Globulins	2.9	2.5	2.74	g/dL	2.3 - 5.3
Albumin/globulin ratio	1.4	1.2	1.8	-	1.0 - 1.5
AST (aspartate aminotransferase)	30	38	28.3	U/L	5 - 34
ALT (alanine aminotransferase)	34	45	39	U/L	0 - 55
Alkaline phosphatase	82	106	83	U/L	40 - 150
Iron	38	120	-	µg/dL	65.00 - 175.00
LDH (lactate dehydrogenase)	204	597	181	U/L	125 - 243
Sodium	137	-	141	mEq/L	136.00 - 145.00
Potassium	3.99	-	3.8	mEq/L	3.50 - 5.10
Chloride	104	-	103	mEq/L	98.00 - 107.00
All parameters measured through kinetic turbidimetric method.					

After the tests were done, the patient improved spontaneously: diplopia and nystagmus disappeared and so did the facial asymmetry and nausea. It was decided that he was to be discharged and asked to come for an office follow-up the next day.

At the office follow-up, low-grade fever (38.1ºC, axillary) and nuchal rigidity were noted. Abducens nerve palsy, nystagmus, facial asymmetry and postural instability had all disappeared. He persisted with singultus, photophobia and nausea. No rash had appeared and the rest of the physical exam was unremarkable. Upon suspicion of meningitis, a neurologist ordered a lumbar puncture that was performed at the emergency room. Opening pressure was 18cmH_2_O.

Cerebrospinal fluid (CSF) analysis did not show leukocytosis, hypoglycorrhachia, hyperproteinorrachia or albuminocytologic dissociation. It did, however, showed a red-blood-cell (RBC) concentration of 20 units per cubic millimeter, suggesting a traumatic tap. Gram, Schiff, Ziehl-Neelsen, India ink, acridine orange and potassium hydroxide (KOH) staining of CSF did not show any bacteria, fungus or parasite. Aerobic, anaerobic and fungal cultures were seeded and put in incubation under standard conditions. A summary of CSF findings can be seen in Table 4.

**Table 4:** Cerebrospinal fluid analysis

Cerebrospinal fluid analysis				
Parameter	Result	Units	Reference values	Test tube
Gross appearance	Clear and colorless with a viscosity similar to that of water; no clots were observed	-	-	Test tube #1
Glucose (cerebrospinal fluid)	65	mg/dL	40 - 80	
Glucose (plasma)	72	mg/dL	65 - 100	
Total proteins	46.73	mg/dL	12 - 60	
Total Leukocytes	0	mm^3^	0 - 5	
Lymphocytes	0	%	-	
Neutrophils	0	%	-	
Red blood cells	20	mm^3^	-	
Non-crenated red blood cells	100	%	-	
Crenated red blood cells	0	%	-	
Gram stain	Negative: no microorganisms were observed	-	-	Test tube #2
Ziehl-Neelsen stain (light microscopy)	Negative: no acid-fast microorganisms were observed	-	-	
Auramine stain (fluorescence)	Negative: no acid-fast microorganisms were observed	-	-	
Potassium hydroxide (KOH) stain	Negative: no fungal structures were observed	-	-	
Schiff stain	Negative: no fungal structures were observed	-	-	
Acridine orange stain	Negative: no microorganisms were observed	-	-	
India ink stain	Negative: no Cryptococcus neoformans was observed	-	-	
Methods: photometry and direct microscopic observation.				

A new complete blood count (CBC) was ordered and did not show any relevant changes with respect to the one taken one day prior (see table 2).

As the clinical features and test results matched the findings commonly seen in aseptic meningitis and no polymerase-chain-reaction (PCR) confirmation was available at the time, outpatient supportive treatment with acetaminophen, ibuprofen and generous hydration was started. After more than 80 hours, singultus finally disappeared. 

Over the following five or six days, the patient improved. Most neurological symptoms disappeared; but he remained with constant low-grade fever (38.1ºC to 38.4ºC), intermittent photophobia, minimal nausea and slight hyperacusis. He wasn’t in any pain and other than the previously listed, and did not report any more symptoms.

Since some of the clinical features were not entirely consistent with the diagnosis of aseptic meningitis, additional tests were ordered. Serologic testing for Epstein-Barr virus, human immunodeficiency virus (HIV), and cytomegalovirus were all negative. A chest radiograph and urinalysis did not report any abnormalities. Cultures seeded from CSF on day 2 had not shown signs of bacterial or fungal development. A new CBC showed a moderate decrease in hemoglobin concentration, total RBCs, hematocrit and platelet count. Total leukocyte count remained mostly unchanged but the differential analysis showed a relative increase of lymphocytes (see table 2). Coagulation profile results were within reference parameters. The concentration of alkaline phosphatase had doubled, total bilirubin had increased slightly, iron concentration had increased 4x, gamma-glutamyltransferase (GGT) was slightly above the reference range. The rest of the assessed parameters were within reference values and can be seen in Table 3. No conclusive diagnosis was made and treatment continued without changes. 

On the ninth day following the initial consultation, the patient developed a new, interesting pattern: for about eight hours he would remain completely asymptomatic and then he would experience a short period (of about 10 minutes) of intense shivering and what he described as “the coldest cold” he had ever felt, followed by very high (up to 40.5ºC) sweatless fever, severe headache, nausea, and vomiting. Then, after 40-80 minutes, the fever would disappear and he would start sweating profusely. Malaise, myalgias, arthralgias and anorexia would appear for some hours and then he would be asymptomatic and feeling well again. Ondansetron and ranitidine were added to acetaminophen, ibuprofen and IV crystalloids.

An infectious disease specialist and a professor of medical microbiology and immunology were consulted. A new physical exam showed splenomegaly, hepatomegaly and scleral icterus. Neurological examination was unremarkable as was the rest of the exam. Testing for dengue, Zika, Chikungunya, hepatitis A and B, CBC, comprehensive metabolic panel and urinalysis were ordered. The new laboratory assessment showed that the patient had developed normocytic, normochromic anemia, thrombocytopenia, hyperbilirubinemia, hematuria, and haemoglobinuria. Viral testing was negative (see table 5).

**Table 5:** Summary of viral testing Abbreviations: ELISA: Enzyme-linked immunoabsorbent assay; CMV: Cytomegalovirus; EBV VCA: Epstein-Barr Virus viral capsid antigen; EBV EAD: Epstein-Barr Virus early antigen diffuse complex; EBV EBNA: Eptstein-Barr Virus nuclear antigen; HAV: Hepatitis A Virus; HIV: Human Immunodeficiency Virus; HBsAg: Hepatitis B virus Australia Antigen; HBsAb: Hepatitis B virus surface antibody; HBeAg: Hepatitis B virus e antigen; HBeAb: Hepatitis B virus e antibody; HBcAb: Hepatitis B virus core antibody; HBV DNA: Hepatitis B virus DNA; RT-PCR: real-time polymerase-chain reaction.

Other tests	
Assessed parameter	Result
CMV IgM (ELISA)	Negative
CMV IgG (ELISA)	Negative
EBV VCA-IgM (ELISA)	Negative
EBV VCA-IgG (ELISA)	Positive
EBV EAD-IgG (ELISA)	Negative
EBV EBNA IgG (ELISA)	Positive
HAV IgG (ELISA)	Negative
HAV IgM (ELISA)	Negative
HIV 1/2 (ELISA) (BIO-RAD Genie ® Fast HIV 1/2)	Negative
HBsAg (ELISA)	Negative
HBsAb (ELISA)	Positive
HBeAg (ELISA)	Negative
HBeAb (ELISA)	Negative
HBcAb (ELISA)	Negative
HBV DNA (RT-PCR)	Negative
Dengue NS1 antigen	Negative
Dengue IgG	Positive
Dengue IgM	Negative
Dengue (RT-PCR)	Negative
Zika (RT-PCR)	Negative
Chikungunya (RT-PCR)	Negative
Zika IgM	Negative
Chikungunya IgM	Negative
All parameters were evaluated from blood serum unless stated otherwise.	

Both experts reviewed the tests and history, and suspected malaria. Thick and thin blood smears were taken and sent to the laboratory for analysis. On the thirteenth day, the diagnosis of *Plasmodium vivax* malaria was made and confirmed by the national reference laboratory. Treatment was started with chloroquine phosphate, following the guidelines from the CDC and the Pan-American Health Organization (PAHO).

Chloroquine treatment resulted in the patient developing severe nausea and frequent vomiting, particularly after the second dose was administered. The patient then developed anorexia, barely tolerating puréed food and cool liquids. Once the last dose of chloroquine was administered, the patient improved slightly.

Primaquine (30mg) was administered daily for 15 days immediately after the chloroquine regime was completed. While primaquine was being administered, vomiting continued intermittently and the patient developed tachypnea and respiratory distress, requiring supplemental oxygen. The patient also developed hyposphagma, thrombocytopenia, non-nephrotic proteinuria, hematuria, and epistaxis. 

In spite of presenting features of severe malaria for which a different treatment regime is suggested, the physicians in charge, following recommendations from specialized personnel dispatched from the infectious disease division from the state and national health authorities, opted to continue with daily primaquine and follow-up blood smears.

From the sixth day after treatment with chloroquine was started (day 21 since the first symptom), no *Plasmodium vivax* blood forms could be visualized in the follow-up blood smears, which continued to be processed bidaily for 21 days. Subsequent follow-up laboratory tests were scheduled at days 30, 60, 90 and 120.

**Figure 1:**
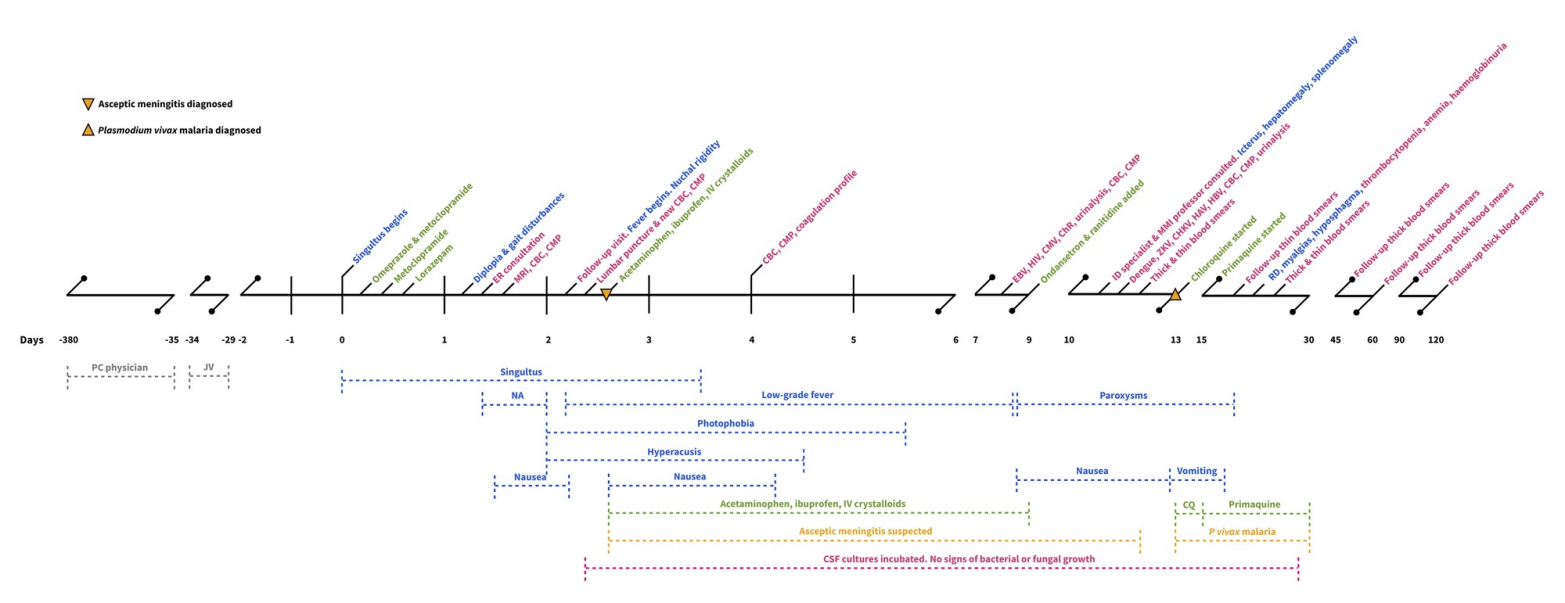
Timeline summarizing relevant facts Timeline highlighting symptoms and physical exam findings (blue), drugs (green), laboratory tests and medical interventions (dark pink), presumptive diagnoses (orange) and general information (gray). Abbreviations: PC physician: primary-care physician; JV: Lacandon jungle visit; ER: emergency room;  MRI: magnetic-resonance imaging; CBC: complete-blood count; CMP: complete metabolic panel; NA: neurological abnormalities (nystagmus, abducens nerve palsy, facial asymmetry, reduced strength, hyperactive reflexes, Romberg’s sign); IV: intravenous; CSF: cerebrospinal fluid; EBV: Epstein-Barr Virus; HIV: human-immunodeficiency virus; CMV: Cytomegalovirus; ChR: chest radiograph; ID: infectious disease; MMI: medical microbiology and immunology; ZKV: Zika virus; CHKV: Chikungunya virus; HAV: Hepatitis A virus; HBV: Hepatitis B virus; CQ: chloroquine; RD: respiratory distress.

The patient suffered from the sequels of malaria: he lost 13kg (almost 20% of his total body weight) and 10g/dL of hemoglobin in the first 20 days since the symptoms began (see a summary of CBC changes in table 2). He recuperated slowly and without permanent sequelae. At the follow-up visit 120 days later, he had recovered fully. He was advised to have biannual follow-up blood smears and to seek prompt medical consultation in case of any febrile disease. A visual summary of the most relevant facts previously described can be seen in Figure 1.

## Discussion

Malaria is a ubiquitous vector-borne disease caused by inoculation of *Plasmodium* sporozoites by the bite of a female *Anopheles* mosquito into a human host [6, 9]. The World Health Organization (WHO) reports that *Plasmodium spp* infections are present in at least 91 countries [9] and in 2017, there were an estimated 219 million cases worldwide [9]. *Plasmodium falciparum* infections represent the largest burden of disease, closely followed by those caused by *Plasmodium vivax*, the predominant species in the Americas [9].

More than a hundred different *Plasmodium* species are recognized [6], but only four infect humans regularly [6, 9]: *P malariae*, *P falciparum*, *P vivax*, and *P ovale*. Recently, reports of human infection with *P knowlesi *[9], *P cynomolgi *[9, 10], and *P simium *[11] have emerged.

Classic malaria symptoms develop once blood parasitemia is achieved after the liver stage is completed [6]. In *P vivax*, this is typically two to three weeks after inoculation [12]. High fever, malaise, fatigue, headache, diaphoresis, chills, diarrhoea, mild abdominal pain, and nausea are frequent manifestations of the disease, overlapping with those of other common tropical diseases such as Dengue, Zika, Chikungunya, typhoid fever, Hepatitis A, leptospirosis, and meningitis [7,13].

As it happens with other diseases, malaria can have unusual presentations: a review of more than 40 years of reported data carried out by Zaki and Shanbag in 2002 showed that the classical presentation -that is, “febrile paroxysms alternating with periods of otherwise relative wellness”- is seen only in 50-70% of all cases [14]. They compiled reports that describe atypical neurological, psychiatric, hematological, ocular, musculoskeletal, renal, respiratory, cardiovascular, gastrointestinal, metabolic, endocrine, cutaneous, and pregnancy-related manifestations of malaria. However, in their exhaustive search, no association was found between singultus and Plasmodia [14]. Another review by Mohapatra et al that specifically described atypical manifestations of *vivax* malaria did not find an association between hiccups and *Plasmodium vivax *[15]. More unusual symptoms such as ageusia, apyrexia, odynophagia, cough and urinary discomfort have been described more recently as the presenting complaints in patients with laboratory-confirmed malaria [16], but singultus has yet to be reported as an associated symptom to *Plasmodium* infections.

Hiccups' afferent impulse is carried by the vagus and phrenic nerves, along with sympathetic nerve fibers of the thoracic region [4]. The efferent response is carried by the phrenic nerve, causing unilateral -and, occasionally, bilateral- myoclonic contractions of the diaphragm [1,4]. Contractions occur with a frequency of 4-60 per minute [1] and have been observed *in utero*, suggesting a possible role in the training of inspiratory muscles [1]. Singultus classification is based on their duration: acute attacks last no longer than 48 hours; ‘persistent singultus’ last more than two days and ‘intractable’ singultus last more than 1 month [1].

Hiccups have been previously associated with a series of different diseases -among them, infections- [1] (see Table 6) that affect multiple organs.

**Table 6:** Previously known singultus associations

Previously known singultus associations [1]	
Disease	Type of insult
Stroke, meningitis, encephalitis, brain abscess, neuromyelitis-optica-spectrum-disorders, Parkinson’s disease, epilepsy, multiple sclerosis	Neurologic
Pneumonia, bronchitis, asthma, rhinitis, otitis, pharyngitis, pharyngeal intubation	Respiratory
Myocardial infarction, pericarditis, myocarditis, pericardial effusion	Cardiac
Intracranial tumors, mediastinal tumors, cardiac tumors, bronchial carcinoma, esophageal carcinoma, gastric carcinoma	Oncologic
Tuberculosis, herpes zoster, bacterial and viral meningitis, encephalitis	Infectious
Trauma, Sjögren’s syndrome, hiatus hernia, electrolyte imbalances, anxiety, excitement, stress, fear, and the administration of some drugs	Other

The mechanism by which singultus is thought to appear as a symptom of an underlying infection is that of direct damage caused by the offending microorganism in any of the components of the proposed reflex arc. In the case of infection-related, centrally-caused singultus, it is reasonable to hypothesize that the microorganisms have to cross the blood-brain-barrier (BBB) for brain damage to occur. Generally, when pathogens manage to traverse the BBB, marked abnormalities can be seen in the CSF analysis. Hypoglycorrhachia, leukocytosis and turbid CSF are traditionally associated with bacterial insults; hyperproteinorrachia and a mild elevation of the leukocyte count is often seen when a virus affects the CNS, and a dyschromic fluid is sometimes found when tuberculosis or fungal agents directly damage the meninges or the encephalus. Harm caused by parasites and other non-bacterial, non-viral, non-fungal agents ordinarily produces nonspecific CSF findings, but even when no microorganisms can be seen in the initial staining of the cerebrospinal fluid, they can be recovered later through specific cultures or specialized testing. There is no known classical pattern of CSF findings in patients with malaria.

Less than 2% of all *Plasmodium* infections result in severe manifestations of the disease, one of which is cerebral malaria [8,17]. The operational definition proposed by the WHO is that of "a clinical syndrome characterized by coma (inability to localize a painful stimulus) at least 1 hour after termination of a seizure or correction of hypoglycemia, detection of asexual forms of *P falciparum* parasites on peripheral blood smears, and exclusion of other causes of encephalopathy" [8]. There is an almost homogenous clinical presentation of cerebral malaria in children, but adults present with a more complex multiorgan syndrome [8]. Headache and delirium typically precede coma and encephalopathy, which can present as dysconjugate eye deviation, extrapyramidal rigidity and trismus [8].

Some of the neurological symptoms previously associated with cerebral malaria were seen in our patient, notably in the absence of all of the three features included in the operational definition of cerebral malaria made by the WHO. On day 1, and throughout the course of the disease, he was able to localize painful stimuli and did not develop seizures at any point. The direct light microscopy of CSF and blood films taken on days 1 and 2 did not evidentiate sexual forms of *Plasmodium* or any other pathogen, and the initial and *ex post facto* investigations reasonably discarded other causes of encephalopathy.

Diagnosing this patient was difficult, as he presented an extremely unusual presentation of both singultus and *Plasmodium vivax* infection. Specific testing for malaria was delayed, partly because it is an exceedingly rare disease in the country where all the events developed, and partly because the classical "tertian" or "quartan" fevers appeared late in the course of the disease (9 days after the initial symptoms).

Some aspects of the way in which medical care was provided to this patient can be contested, particularly the decision to discharge and start outpatient management on day 1. That resolution was taken to try to accommodate the patient's wishes, who happened to be an strong-willed physician. One of the considerations that the medical team made at the time was that he was reasonably well trained to promptly identify complications and seek appropriate assistance, if needed. The fact that the diagnosis of aseptic meningitis was based entirely on clinical features, without confirmation by PCR and with a dubious interpretation of laboratory tests was also influenced by the patient participating actively, in his role as a physician, in his own medical attention. In hindsight, this may not have been the best choice, as it delayed testing of some parameters that he thought were unnecessary.

## Conclusions

Singultus is a common symptom, usually benign in nature and without further complications. Malaria is a very common disease worldwide (but infrequent in the patient's country) and its typical presentation is that of a febrile disease with many non-neurological symptoms. When Plasmodia do produce neurological symptoms, they are often severe and combined with other clinical manifestations of multiorgan damage. No previous reports of *Plasmodium vivax* and singultus have been described and the mechanism by which it could be caused in this case remains elusive, but is probably different to that of cerebral malaria, as the patient did not meet the classification criteria and did not develop other clinical features. This case is relevant because it documents for the first time an atypical presentation of one common symptom and one frequent disease and describes this previously unreported association. Further study is needed to understand thoroughly all the clinical implications.
